# Sirtuins and SIRT6 in Carcinogenesis and in Diet

**DOI:** 10.3390/ijms20194945

**Published:** 2019-10-07

**Authors:** Maria de Céu Teixeira, Elena Sanchez-Lopez, Marta Espina, Maria Luisa Garcia, Alessandra Durazzo, Massimo Lucarini, Ettore Novellino, Selma B. Souto, Antonello Santini, Eliana B. Souto

**Affiliations:** 1 Department of Pharmaceutical Technology, Faculty of Pharmacy, University of Coimbra (FFUC), Polo das Ciências da Saúde, Azinhaga de Santa Comba, 3000-548 Coimbra, Portugal; mceuteixeira1@gmail.com; 2 Department of Pharmacy, Pharmaceutical Technology and Physical Chemistry, Faculty of Pharmacy and Food Sciences, University of Barcelona, Av. Joan XXIII 27-31, 08028 Barcelona, Spain; esanchezlopez@ub.edu (E.S.-L.); m.espina@ub.edu (M.E.); marisagarcia@ub.edu (M.L.G.); 3 Institute of Nanoscience and nanotechnology (IN2UB), Faculty of Pharmacy, University of Barcelona, 08028 Barcelona, Spain; 4 CIBERNED Centros de Biomedicina en Red de Enfermedades Neurodegenerativas, Facultat de Farmàcia, Universitat de Barcelona, 08028 Barcelona, Spain; 5 CREA—Research Centre for Food and Nutrition, Via Ardeatina 546, 00178 Rome, Italy; alessandra.durazzo@crea.gov.it (A.D.); massimo.lucarini@crea.gov.it (M.L.); 6 Department of Pharmacy, University of Napoli Federico II, Via D. Montesano 49, 80131 Napoli, Italy; ettore.novellino@unina.it; 7 Department of Endocrinology of Hospital de São João, Alameda Prof. Hernâni Monteiro, 4200–319 Porto, Portugal; sbsouto.md@gmail.com; 8 CEB—Centre of Biological Engineering, University of Minho, Campus de Gualtar, 4710-057 Braga, Portugal

**Keywords:** sirtuins, SIRT6, cancer, modulator, chemotherapy, diet

## Abstract

Sirtuins are a highly conserved family of nicotinamide adenine dinucleotide (NAD)-dependent protein lysine modifying enzymes. They are key regulators for a wide variety of cellular and physiological processes such as cell proliferation, differentiation, DNA damage and stress response, genome stability, cell survival, metabolism, energy homeostasis, organ development and aging. Aging is one of the major risk factors of cancer, as many of the physiological mechanisms and pathologies associated with the aging process also contribute to tumor initiation, growth and/or metastasis. This review focuses on one the mammalian sirtuins, SIRT6, which has emerged as an important regulator of longevity and appears to have multiple biochemical functions that interfere with tumor development and may be useful in cancer prevention and for site-specific treatment. The recent evidence of the role of SIRT6 in carcinogenesis is also discussed, focusing on the potential use of SIRT6 modulators in cancer nanomedicine.

## 1. Introduction

Aging is a natural process depending on time, where life progresses and cells experience maturation leading to senescence and death. It causes a decline of the normal functioning of organ systems, increasing the incidence of age-related diseases such as diabetes, cardiovascular and neurodegenerative diseases and cancer [1].

In general, cancer pathophysiology is marked by genomic instability and altered cell metabolism. Genomic instability seems to facilitate the origin of tumorigenic mutations that supports tumor cell survival and proliferation and can be caused by genotoxic agents (e.g., ionizing radiation or ultra-violet radiation) or metabolic reactive oxygen species (ROS) inducing DNA damage. To overcome this issue and maintain genomic integrity, eukaryotic organisms have developed highly coordinated cellular DNA damage response. In addition, tumor cells metabolism is different from normal and healthy cells, being characterized by elevated aerobic glycolysis, known as the Warburg effect [2], enhanced glutamine metabolism [3] and alterations in lipid metabolism [4]. These two events seem to be interconnected, as DNA damage may induce alterations in metabolism and several metabolic proteins that intervene in glycolysis and glutaminolysis are important pieces in cell cycle regulation and genomic repair [5,6,7]. Therefore, the molecules that regulate metabolism in response to genomic instability and damage may help in the understanding of tumorigenesis and serve as potential targets in new cancer therapy strategies [8].

The protein acetylation is a post translational reaction that regulates cellular processes such as DNA recognition, protein-protein interaction, catalytic activity and protein stability [9,10]. Acetylation and deacetylation reactions at lysine residues are catalyzed by histone acetyltransferases [11] and histone deacetylases [11]. HDACs are classified in four classes (I-IV) [11]. Classified as Class III HDACs, sirtuins are a family of proteins, present in eukaryotic organisms, conserved throughout generations that are homologous to Silencing Information Regulator *Sir2* found in yeasts. These proteins perform deacetylase and/or mono-ADP (adenosine di-phosphate)-ribosyltransferase activities requiring the cellular metabolite NAD+ (nicotinamide adenine dinucleotide), interfering in many important cellular processes including regulation of metabolic pathways, cell regulation and surviving, aging and inflammation [12,13]. Mammalian sirtuins are divided in seven family members (SIRT1-7, Table 1). SIRT1 and SIRT2 are localized in the nucleus and cytoplasm. SIRT3, SIRT4 and SIRT5 are mitochondrial, and SIRT6 and SIRT7 are nuclear [14].

SIRT6 is one of the mammalian sirtuins that have gotten the most interest in several studies in recent years, and through its effects on histone deacetylation, SIRT6 protects against aging and age-related diseases, as among which cancer [15]. SIRT6 deacetylase activity occurs preferably in H3 lysine 9 (H3K9) [16] and H3K56 [17,18], which relate to the promotion of genomic stability, telomere maintenance and modulation of gene expression, interfering, for instance, in secretion rates of tumor necrosis factor α (*TNFα*) (as discussed below). Additionally, evidence shows that SIRT6 is implicated in the regulation of aerobic glycolysis in tumor cells. This double role in genomic stability and in metabolism can be translated into considering that SIRT6 has a tumor regulation activity [19].

Therefore, due to this important role in tumorigenesis, selective modulation of SIRT6 may be pharmacologically interesting, especially because of the potential application in therapeutic and chemo-preventive strategies [20,21]. There is a growing interest in understanding the regulatory genomic and metabolic nodes that can be targeted in cancer treatment and determining whether sirtuins specifically may be promising biomarkers or therapeutic targets in cancer [22].

This review addresses the sirtuins role in cancer pathophysiology, with a special interest of the SIRT6 action in different kinds of cancers, as well as the first efforts in SIRT6 modulators research.

## 2. Sirtuins and Cancer

These sirtuins are a conserved family of proteins with deacetylase and/or mono-ADP ribosyltransferase NAD+ dependent activities [12]. These proteins are the mammalian homologous to the silencing information regulator *Sir2*, a silencing factor found in *Saccharomyces cerevisiae* [23] that promotes longevity in yeasts, being a key factor in transcriptional gene silencing through regulation of histone modifications [24,25].

Mammalian sirtuins are specific histone deacetylases and also class III HDACs that are divided in seven types (SIRT1-7). The members of sirtuins family differ widely in their cellular localization, catalytic activity, and functions. SIRT1, 6 and 7 are localized in the nucleus; SIRT2 and SIRT3 are mainly cytoplasmic and mitochondrial, respectively, while present in limited levels in the nucleus; SIRT4 and 5 are found strictly in mitochondria [26,27,28]. The localization of sirtuins can also depend on the cell function or tissue type, stage of development, metabolic status and certain stress conditions. These factor are also correlated to their function regulation [29].

These molecules have been widely studied in terms of their catalytic properties, substrates, and molecular functions. Besides lysine residues deacetylation (the first established activity of these enzymes), recent in vitro evidence indicates that many sirtuins can catalyze longer chain deacetylations, including the removal of hexanoyl, octanoyl, decanoyl, dodecanoyl, myristoyl and palmitoyl groups. These results might explain the broad diversity of sirtuins in biological functions [30]. It has been recently described that interconversion of cytosolic phosphoenolpyruvate carboxykinase (PCK1) between gluconeogenic and anaplerotic activities is attributed to its dynamic acetylation [31].

Sirtuins perform many functions such as chromatin regulation, cell survival under stress, metabolic homeostasis regulation, cellular development, differentiation and senescence, which also implicate multiple cellular processes including transcription, metabolism, fat mobilization, DNA repair and apoptosis; these are key factors, for instance, in tumorigenesis and aging [32,33]. With sirtuins playing an important role at cellular and organic levels, they have been linked to cancer, diabetes, cardiovascular diseases and neurodegenerative diseases, among other age related diseases [24].

Sirtuins may apparently play a dual role in cancer. On the one hand, some sirtuins seem to help to protect DNA (from damage and oxidative stress), maintain genomic stability and limit replicative life span (which suggests that they can protect organisms against cancer); on the other hand, some data suggest that cell survival under stress conditions promoted by sirtuins may be directly involved in tumorigenesis, as it would inhibit senescence allowing undifferentiated cell proliferation [29]. Additionally, a number of studies have shown that sirtuins also regulate the nutrient state of a tumor. Sirtuins mediate control of metabolic pathways with a focus on glucose metabolism, the tricarboxylic acid (TCA) cycle and against reactive oxygen species (ROS) in cancer [22].

As the focus of this review is mainly SIRT6, we refer to Cha et al. [34] for in vitro and in vivo experimental evidence on the different roles of all types of sirtuins in cancer. For a better understanding of carcinogenesis events and mechanisms in which these enzymes are involved, we also refer to Jeong et al. [8] and German et al. [22].

## 3. SIRT6: Oncogene or Tumor Suppressor?

**Figure 1:**
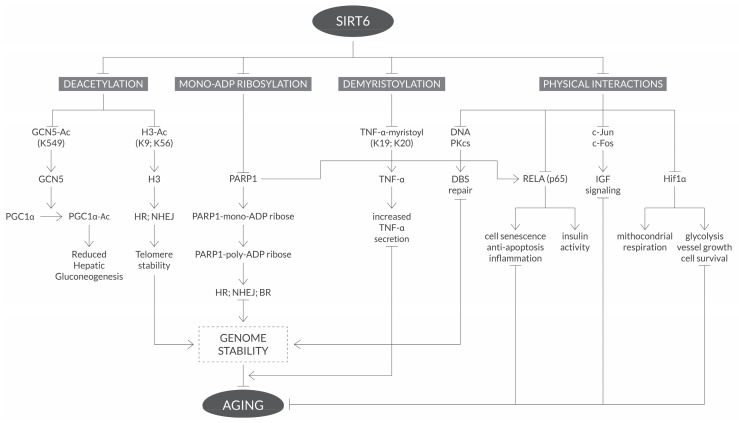
SIRT 6 activities and functions. SIRT6 described activities, substrates and responses related to the aging process.

SIRT6 is a member of human sirtuin sub-class IV. Similar to other sirtuin structures, SIRT6 has a two-domain structure that consists of a large Rossmann fold and a smaller and structurally more variable sequence containing a Zn^2+^-binding motif. SIRT6 activities are both deacylation of acetyl groups and long-chain fatty acyl groups such as myristoyl, and as a mono-ADP-ribosyl transferase. Deacetylation of lysine by SIRT6 is coupled to NAD+ hydrolysis, yielding *O*-acetyl-ADP ribose and nicotinamide. The nicotinamide product of this reaction is thought to allosterically inhibit SIRT6 activity [20]. Among the seven mammalian sirtuins, SIRT6 is a critical regulator of diverse processes, including DNA repair, gene expression, telomere maintenance, metabolism and aging [35]. SIRT6 deficiency or inactivation in mice, results in shortened life spans and degenerative phenotypes [36]. SIRT6 deficiency in mice is characteristically dominated by metabolic defects, such as severe hypoglycemia, lymphocytic apoptosis and wasting [37]. SIRT6 knockdown cells, or those where the level of SIRT6 expression is reduced, undergo premature cellular senescence, exhibiting genetic defects such as telomere dysfunction and chromosomal end-to-end fusion [38]. On the other hand, overexpressing exogenous SIRT6 male mice have a significantly longer lifespan than the wild-type mice [12]. This evidence shows SIRT6 regulatory activity, linking energy homeostasis to lifespan, and shows that aging is presented as one of the major risk factors of cancer. This same activity may be considered a key factor in tumorigenesis. In fact, recent studies have proven that SIRT6 plays an important role in cancer related cellular pathways and processes such as metabolism, genome stability, cellular proliferation and apoptosis, inflammation and immunocompetence (Figure 1).

Many of the diverse functions of SIRT6 are carried out by its deacetylation activity of H3K9 [39] and H3K56 [40] histone marks and different cellular proteins. Histone deacetylation is associated with heterochromatin formation and decreased chromatin accessibility. Histone deacetylase activity of SIRT6 is nucleosome dependent. Only when histones H3 and H4 are packaged as nucleosomes, SIRT6 associates with the nucleosome and deacetylates at high efficiency. Other substrates that are deacetylated by SIRT6 are the double strand break resection protein *CtIP* (which is the C-terminal binding protein (*CtBP*) interacting protein) and the histone acetyltransferase GCN5. SIRT6 has also been shown interaction with mono-ADP-ribosylate poly(ADP-ribose) polymerase 1 (*PARP*1) to regulate DNA repair in response to genotoxic stresses [41].

SIRT6 deacetylation activity can generate responses in metabolic processes and pathways [42]. SIRT6 interaction with hypoxia inducible factor-1α (*HIF1α*) and the deacetylation of H3K9 at the promoter of *HIF1α* target genes was found to suppress directly the expression of multiple glucose-metabolic genes. *HIF1α* is known to modulate multiple genes to activate glycolysis and repress simultaneously mitochondrial respiration. Zhong et al. [43] validated the SIRT6-mediated inhibition of *HIF1α* in vivo, where the treatment of SIRT6-deficient mice with a *HIF1α* inhibitor rescued the hypoglycemic phenotype [43]. SIRT6 can also control gluconeogenesis through activity modulation of peroxisome proliferator-activated receptor-α coactivator 1α (*PGC-1α*), which is the main regulator of gluconeogenesis that stimulates hepatic gluconeogenesis in part by increasing the expression of gluconeogenic enzymes [44]. Evidence points to another non-histone substrate for SIRT6, namely GCN5, and have uncovered a SIRT6-mediated pathway for the control of *PGC-1α* activity and hepatic glucose production [45]. SIRT6 interacts with GCN5 (general control non-repressed protein 5), which enhances its activity. GCN5, in turn, is an acetyltransferase that catalyzes acetylation of *PGC-1α*. Other work demonstrated that SIRT6 overexpression reduced gluconeogenic gene expression in the liver of wild type but not liver-specific forehead box O1 (FoxO1)/3/4 knockout mice [46]. Moreover, long-chain deacylase activity was found to be an intrinsic activity of most sirtuins [47]. In fact, it was also found that SIRT6 can hydrolytically remove long-chain fatty-acyl groups, including myristoyl and palmitoyl groups, in a process known as lysine deacylation [48].

Cancer development may also be related to chronic inflammation, since the malignant microenvironment supplies growth factors that contribute to cancer cell proliferation, angiogenesis, invasion, metastasis and signals, leading to epithelial mesenchymal transition (EMT). One of the key factors involved in inflammation-mediated cancers is the tumor necrosis factor alpha (*TNFα*), which is implicated in selective destruction of tumor blood vessels and hemorrhagic necrosis. When produced in the microenvironment of the tumor, *TNFα* may also act as tumor promoter. At a post-transcriptional level, SIRT6 regulates *TNFα*, connecting metabolism and inflammation. SIRT6 promotes *TNFα* secretion from the cell by means of demyristoylation (i.e., removal of myristoyl modifications from lysine 19 and 20 of *TNFα* by deacylation of long chain fatty acids) [49]. As a result, inhibition of SIRT6 may be useful against cancer-induced inflammation, angiogenesis and metastasis [50].

*NF-κB* is a protein complex involved in the control of DNA transcription, cytokine production and cell survival. *NF-κB*, which is activated by *TNFα*, is involved in the initiation and progression of cancer. Its targets are involved in inflammation, cell proliferation, angiogenesis, survival, invasion, metastasis and in EMT [51,52]. It has also been described that SIRT6 may act as an anti-inflammatory enzyme by controlling *NF-κB*-dependent gene expression. SIRT6 binds to *NF-κB* RELA subunit and deacetylates H3K9 at *NF-κB* target gene promoters, leading to repression of *NF-κB* signaling.

The overexpression of SIRT6 potentiates apoptosis in a series of cancer cell lines, but not in normal, non-transformed cells. This event is not mediated by the activity of deacetylase of SIRT6, but instead by the activity of the mono-ADP ribosyl transferase. Apoptosis promoted by SIRT6 occurs via p53 or p73 signaling [53], and it needs ATM kinase to initiate the response [5]. Survivin is a prosurvival protein belonging to the inhibitor of apoptosis (*IAP*) family. It blocks apoptosis by inhibiting apoptosis inducing factor (*AIF*) dependent apoptotic pathways and eventually caspases-mediated apoptosis. While diffusedly expressed during tissue development, in most normal tissues, the protein is absent or expressed at very low levels. In most of the cancers, the expression of survivin is re-activated, which is associated with tumor aggression and a decrease in patient survival rates. The expression of survivin was shown to be controlled by SIRT6, which is involved in the modulation of histone deacetylation and *NF-κB* binding at the survivin promoter. SIRT6 expression is regulated by *c-Fos* and, by repressing surviving, it potentiates cell death. The inhibition of *c-Fos* by c-Jun in early tumor stages blocks SIRT6 expression. SIRT6 repression of survivin has also been demonstrated in cells with lower expression of SIRT6 as those of the endometrial cancer. Targeting the anti-apoptotic activity of survivin at the initiation stage (or increase of the levels of SIRT6) may compromise the development of cancer which may be used as an approach against tumor set-up and age-related diseases. The SIRT6 expression is also involved in regulation of metabolic homeostasis and in genomic integrity. In cancer pathophysiology, it may have a dual effect, i.e., either tumor suppressor or as oncogene. However, in carcinogenesis SIRT6 activity is dependent on the tissue. SIRT6 regulation can also interfere with chemotherapy sensitization. Therefore, a case is made to map the SIRT6 activity in different cell and tissue localization, such as vascular cells [54,55], bone cell lines [56,57], dental pulp cells [58], the retina [59], heart tissue [60,61] and renal tissue [62,63].

### 3.1. Breast Cancer

Evidence has been given to the survival of breast cancer patients with the abundance of SIRT6 and inversely correlated with induced protein degradation by chemical alterations in SIRT6 specific residues.

Thirumurthi et al. [59] conducted a study in various breast cancer cell lines and a panel of breast tumor biopsies. This study first established a degradation pathway of SIRT6 that starts with the phosphorylation at Ser388 of the molecule by kinase *AKT1*. This event then triggered the ubiquitination of SIRT6 by *MDM2*, marking it for protease-dependent degradation. Exploring this, in a panel of breast tumor biopsies, the results showed that inhibition of *AKT* or preventing SIRT6 phosphorylation by Ser338 mutation prevented the SIRT6 degradation mediated by *MDM2*, suppressed breast cancer cells proliferation in culture, and inhibited the growth of breast tumor xenografts in mice. For the confirmation of these results, the authors evaluated the relationship between *MDM2* expression and the presence of SIRT6. Overexpressing *MDM2* decreased the abundance of SIRT6 in cells, whereas overexpressing an E3 ligase–deficient *MDM2* or knocking down endogenous *MDM2* increased SIRT6 abundance. Furthermore, in a second phase of the study, trastuzumab sensitivity in a resistant breast cancer cell line was correlated to SIRT6 abundance in cells. Trastuzumab targets a specific receptor common in some breast cancers types, and the results showed that SIRT6 knockdown increased the survival of a resistant breast cancer cell line exposed to trastuzumab. On the other hand, the overexpression of a non-phosphorylatable SIRT6 mutant increased this sensitivity. While the biochemical role of SIRT6 in breast cancer is still not completely established, this study came to conclusion that stabilizing SIRT6 may be a clinical strategy to overcome trastuzumab resistance in breast cancer patients, therefore increasing their survival prognostics [64]. In a similar study, the role of SIRT6 in both epirubicin and paclitaxel resistance in breast cancer was identified. Khongkow et al. [65] found that SIRT6 protein levels were elevated in paclitaxel and epirubicin resistant MCF-7 cells. SIRT6 knockout sensitized cells to both paclitaxel and epirubicin, whereas SIRT6 overexpression led to increased resistance to these drugs. This role in paclitaxel and epirubicin sensitivity was found to be via targeting FoxO proteins. The tumor suppressor *FoxO3a* increases its levels of acetylation in mouse embryonic fibroblasts depleted of SIRT6, whereas its induction by epirubicin is attenuated in breast cancer cells overexpressing SIRT6 [65].

### 3.2. Endometrial Cancer

Fukuda et al. [66] elucidated the tumor suppressive role of SIRT6 in endometrial cancer cells. The expression of SIRT6 was shown to negatively affect the proliferation of AN3CA and KLE endometrial cancer cells. Increased expression of SIRT6 resulted in the induction of apoptosis by repressing the expression of the anti-apoptotic protein survivin. Consistent with these results, a survivin inhibitor *YM155* efficiently inhibited cellular proliferation and induced apoptosis. These results revealed that SIRT6 might function as tumor suppressor of endometrial cancer cells. However, there is no significant data that could relate the SIRT6 expression with endometrial cancer type or prognostic [67].

### 3.3. Ovarian Cancer

The ovarian aging is characterized by a gradual decrease in both the number of follicles and the quality of oocytes, while the ovarian reserve is translated by the number of primordial follicles. Zhang et al. [63] explored the relationship between ovarian function and sirtuin expression, by assessing ovarian reserve in mice of different ages and mice subjected to caloric restriction and chemotherapy, counting primordial follicles and determining the expression levels of SIRT1, SIRT3 and SIRT6 in ovarian cells. A decline in the number of primordial follicles was observed in aging mice and mice subjected to chemotherapy. In caloric restriction mice, results showed the contrary. The expression levels of SIRT1, SIRT3 and SIRT6 were also assessed by Western blot analysis, being significantly decreased in the ovaries of aged mice and mice treated with chemotherapy, but increased in caloric restriction mice. These results indicate that SIRT1, SIRT3 and SIRT6 are closely related to ovarian reserve, suggesting that these sirtuins may be markers of ovarian aging [68]. However, additional evidence for the role of SIRT6 as a tumor suppressor in ovarian cancer has also been published, showing that SIRT6 expression is significantly reduced in human ovarian cancer tissues compared to normal tissues. The anti-proliferative effect of SIRT6 on ovarian cancer cells is related to Notch 3 signaling pathway, which is involved in tumor progression of ovarian carcinoma. Indeed, SIRT6 overexpression inhibited the proliferation of ovarian cancer cells, whereas SIRT6 downregulation enhanced cell growth. SIRT6 overexpression was shown to reduce the expression of Notch 3, both at the mRNA and protein levels. However, Notch 3 overexpression blocked this anti-proliferative effect of SIRT6 in ovarian cancer cells [69].

### 3.4. Brain Cancer

SIRT6 may play a role in synaptic function and neuronal maturation and it may be implicated in the regulation of neuronal survival [70] and preservation pathways [71]. Furthermore, in human glioma cells, evidence shows that SIRT6 acts as tumor suppressor. In recent studies, SIRT6 protein and mRNA levels were shown to markedly downregulate when compared to those in normal brain tissues. SIRT6 overexpression also repressed glioma cell growth while SIRT6 knockdown contributes to its growth. *PCBP2* is a member of the *PCBP* family that regulates tumor development and is actively involved in posttranscriptional and translational regulation by interacting with single-stranded poly(C) motifs in target mRNAs [72]. SIRT6 inhibits *PCBP2* expression by binding to *PCBP2* promoter region and deacetylates H3K9, acting, therefore, as a tumor suppressor. SIRT6 expression was negatively correlated with the expression of *PCBP2* (Poly(rC)-binding protein 2.

### 3.5. Liver Cancer

SIRT6 expression was downregulated in human hepatocellular carcinoma (HCC) cells and tissue compared to adjacent normal tissue. SIRT6 knockdown promoted growth of the HepG2 HCC cell line, whereas SIRT6 overexpression inhibited the growth of HepG2 cells inducing cell apoptosis. In addition, SIRT6 overexpression decreased intracellular reactive oxygen species and superoxide anion levels and inhibited phosphorylation of extracellular signal-regulated kinases. These data suggested that SIRT6 is a tumor suppressor in HCC cells [73]. The role of SIRT6 in liver cancer tumorigenesis and prognostics has also been recently described. Using genetic mouse models specific for liver cancer initiation, Min et al. [74] have described a regulatory network that connects stress response and histone modification in liver tumor initiation. The survival of initiated cancer cells was controlled through *c-Fos*-mediated apoptosis suppression. In more detail, *c-Fos* induces SIRT6 transcription, repressing survivin throughout histone H3K9 acetylation and *NF-κB* activation. The results of this work showed that increasing the level of SIRT6 or targeting the anti-apoptotic activity of survivin at the initiation stage markedly impairs cancer development [74]. Considering the negative effect of SIRT6 on cellular senescence implies that it may also have the potential to promote tumor development. Following this, Feng et al. [75] reported that the upregulation of SIRT6 expression was required for transforming growth factor (*TGF*)-β1 and H2O2⁄HOCl reactive oxygen species (ROS) to promote the tumorigenicity of hepatocellular carcinoma (HCC) cells. Additionally, a recent study showed that miR-122, the most prevalent type of miRNA in liver cells, and SIRT6 negatively regulate each other’s expression. SIRT6 downregulates miR-122 by the H3K56 deacetylation mechanism. This interplay is manifested in two relevant ways in the liver: firstly, an opposite regulation of a similar set of molecules, and secondly, a loss of a negative correlation between SIRT6 and miR-122 expression is significantly associated with better prognosis in HCC patients. These results can also indicate that the SIRT6-miR-122 correlation may serve as a biomarker for HCC prognosis [73].

### 3.6. Lung Cancer

A similar effect was also found in other cancer types. The levels of SIRT6 are decreased in non-small cell lung cancer (NSCLC) samples. SIRT6 overexpression in human NSCLC cell lines inhibited their proliferation, whereas SIRT6 knockdown promoted it [74]. Additionally, the predominantly cytoplasmic localization of SIRT6 expression was correlated with poor prognosis and reduced chemosensitivity in patients with NSCLC, indicating that SIRT6 could be a useful prognostic marker for this type of cancer [76]. In work by Han et al. [77], a MTT assay showed that overexpression of SIRT6 could inhibit the proliferation in NSCLC cells, whereas SIRT6 knockdown using small interfering RNA promoted NSCLC cells proliferation. On a molecular level, the authors found that SIRT6 suppressed NSCLC cells proliferation via downregulation of *Twist1* [74]. *Twist1* is also known to induce epithelial–mesenchymal transition (EMT) and it is considered to be a key factor in the promotion of metastasis of cancer cells. Evidence showed that SIRT6 degradation was mediated via the PKA (protein kinase A)-dependent inhibition of the Raf-MEK-ERK pathways. Reduced SIRT6 expression potentiates γ-ray-induced apoptosis of NSCLC cells, suggesting that SIRT6 may have oncogenic activity in lung cancer. In addition, in NSCLC cell lines cultured in vitro, with an over-expressed SIRT6 by an adenovirus vector, it has been proven to improve the radiosensitization effect on A549 cells, which can reduce the ability of cell proliferation, change the cell distribution of cell cycle and induce the cell apoptosis. This approach might be an adjuvant therapy in the local treatment of advanced NSCLC [78].

### 3.7. Skin Cancer

An oncogenic role of SIRT6 in human skin squamous cell carcinoma (SCC) has been recognized. Clinically, SIRT6 levels were higher in SCC samples compared to healthy samples. Evidence shows that SIRT6 acts an oncogene in the skin by COX-2 [79] upregulation, an enzyme involved in inflammation, proliferation and cell survival. SIRT6 promoted expression of COX-2 by repressing *AMPK* signaling, thereby increasing cell proliferation and survival and in the skin epidermis. Additionally, SIRT6 expression in skin keratinocytes was increased by exposure to UVB light through activation of the *AKT* pathway [79].

## 4. Future Prospects on the Use of SIRT6 Modulators in Carcinogenesis

Sirtuin research has revealed interconnected metabolic and signaling pathways implied in tumor biology and can be specifically targeted in the cancer treatment development [22]. As the evidence here discussed shows, sirtuins expression can be considered as a metabolic biomarker to potentially designate promising therapeutic approach strategy. Since the discovery of the sirtuin family [80], there has been an effort in research on the catalyzed deacetylation reaction, with three primary focuses being the elucidation of its sophisticated catalytic mechanisms, the clarification of its biological and pathophysiological functions, and the exploration of the therapeutic molecules that can target this enzymatic reaction. The ultimate goal of these efforts is, therefore, to develop novel therapeutic agents, inhibitors or activators of the sirtuin-catalyzed deacetylation reaction [81]. A novel SIRT6 modulator with a lysine-based structure has been identified recently [82], exhibiting the capacity to increase the activity of glycolytic enzymes. Flavonoids have been reported to modulate the activity of SIRT6 [83].

Regarding SIRT6, and as its biological functions are clarified, there has been active research on developing chemical modulators for this enzyme. These molecules are not only explored as potential therapeutic agents, but also as help to elucidate pharmacological features of SIRT6 enzyme and/or the metabolic and signaling pathways in which it interferes. Additionally, the research in this matter is very recent, and these efforts are presented and discussed as follows.

The first study that we refer to concerning SIRT6 deacetylation inhibitors also claims to be the first one that collected SAR data of SIRT6. Kokkonen et al. [84] analyzed the molecular interactions between different molecules and the enzyme, using a homology model. They reported three compounds performing 62−91% SIRT6 inhibition at a 200 μM concentration. Additionally, to allow the development of small molecules that can regulate sirtuin activity, high throughput empirical assays for sirtuins were developed; for example, a fluorogenic assay with 7-amino-4-methylcoumarin (AMC)-myristoyl peptide [85], which is based on the discovery that SIRT6 is a defatty-acetylase (removing long chain fatty acyl groups). However, the fluorogenic peptide containing AMC was considered the cause of false positive hits from screenings, and a new assay was carried out by Li et al. [86] to overcome this limitation. The authors have developed an alternative method called a FRET-based assay suitable for screening SIRT6 modulators, which they claim will be more reliable since no AMC group is present in the new assay protocol.

Singh et al. [87] developed an H3K9 deacetylation guided assay with SIRT6 coated magnetic beads (SIRT6-MB). Using this protocol, they reported the identification of quercetin and vitexin as SIRT6 inhibitors, and studied structurally related flavonoids, including luteolin, kaempferol, apigenin and naringenin [87]. The results obtained in this work allowed the definition of a preliminary pharmacophore for the quercetin binding site on SIRT6, containing three hydrogen bond donors and one hydrogen bond acceptor, which was refined in other study an additional 12 quercetin analogs [88].

Kokkonen et al. [89] evaluated the potential of SIRT6 to deacetylate a set of five fluorogenic substrates based on the H3K56 deacetylation site, and studied nicotinamide and 15 other small molecule sirtuin modulators using the H3K56 based substrate. EX-527, quercetin and three pseudopeptidic compounds were found to be the most potent SIRT6 inhibitors, exhibiting over 50% deacetylation inhibition. These findings describe the first modulators of SIRT6 activity at the H3K56 deacetylation site [89].

He et al. [90] tested thiomyristoyl peptides cell permeation and SIRT6 inhibition in live cells, based on fatty acetylation of *TNFα*; more specifically, they examined whether the thiomyristoyl peptides could increase the lysine fatty acylation level of *TNFα*. *TNFα* from the cells treated with the four SIRT6 inhibitors had increased fluorescent labelling compared to *TNFα* from the control cells, suggesting that these thiomyristoyl peptides could inhibit SIRT6 in cells, especially the BHJH-TM3 peptide. These results revealed that theses inhibitors should be promising starting points for the development of more specific and more potent inhibitors for SIRT6.

An in silico design approach for SIRT6 modulators screening was reported, in order to identify novel and selective scaffolds that would bind and inhibit SIRT6 selectively. The results obtained show several drugs with in vitro activities. The most promising leads show micromolar IC50s have significant selectivity for SIRT6 versus SIRT1 and SIRT2, and are active in cells, as shown by increased acetylation at SIRT6 target lysines on histone 3, reduced *TNFα* secretion, *GLUT-1* upregulation and increased glucose uptake. Taken together, these results show the value of these compounds as starting leads for the development of new SIRT6-targeting therapeutic agents [91,92,93].

With the developed assay referred above for the identification of quercetin, naringenin and vitexin as SIRT6 inhibitors, Singh et al. [94] continued their work trying to identify novel active compounds from *T. foenum graecum* seed extract. Thus, this group reported the application of the SIRT6-MB for ‘fishing’ experiments in a complex matrix, in which orientin and 17 other compounds were identified as SIRT6 binders. This was the first use of a method for ‘fishing’ out active ligands from a botanical matrix [94].

SIRT6 is highly expressed in human breast tissue, and as referred previously, it also mediates resistance to cytotoxic agents and prevents cell differentiation. For this reason, SIRT6 may constitute an attractive target for the development of new anticancer agents to be used alone or in combination with chemo or radiotherapy. Sociali et al. [92] reported the identification of novel quinazolinedione compounds with inhibitory activity on SIRT6. The identified new SIRT6 inhibitors increased histone H3 lysine 9 acetylation, reduced TNF-a production and increased glucose uptake in cultured cells. In addition, these compounds exacerbated DNA damage and cell death in response to the *PARP* inhibitor olaparib in *BRCA2*-deficient Capan-1 cells and cooperated with gemcitabine to the killing of pancreatic cancer cells. Therefore, this new quinazolinedione-based SIRT6 inhibitors could potentially find therapeutic applications as adjuvants in cancer treatment.

In the most recent study to date, Rahnasto-Rilla et al. [95] evaluated a series of *N*-acylethanolamines (NAEs), quercetin and luteolin SIRT6 activity regulation. NAEs increased SIRT6 activity, with oleoylethanolamide having the strongest effect (EC50 value of 3.1 μm) [95,96]. Quercetin and luteolin were demonstrated to have dual functionality with respect to SIRT6 activity; namely, they inhibited SIRT6 activity with IC50 values of 24 and 2 μm, respectively, and stimulated SIRT6 activity more than six-fold (EC50 values of 990 and 270 μm, respectively). The identification of NAEs as SIRT6 activators opens a novel mechanism of action for these molecules as it expands the portfolio of potential SIRT6 modulators.

Another aspect to highlight is the association between bioactive compounds, sirtuins and diet. Bioactive compounds activate similar molecular targets such as Sirt1. It is worth mentioning the work of Pallauf et al. [97], which summarized sirt1-inducing plant bioactive compounds and the foods they are found in: hydroxytyrosol in olive oil, resveratrol in red grapes, quercetin in onions, apples and capers, kampferol in cabbage, kale and parsley, and many others. The same authors suggest that a so-called “MediterrAsian” diet combining sirtuin-activating foods which can be called “sirtfoods”, belonging to the Asian as well as Mediterranean diet may be a promising dietary strategy in preventing chronic diseases, thereby ensuring health and healthy ageing. This opens a new frontier towards nutraceuticals containing these bioactive compounds and adopting a healthy diet [98,99,100,101,102,103,104,105]. Notwithstanding this, and in line with the existing studies on follow up, use and compliance of pharmaceuticals, as shown by recent research in the area [106,107,108], a similar approach, which includes communication strategies and assessment [109], would be necessary to exploit the potential of sirtfoods in the prevention of age-related diseases. This will guarantee proper and scientifically substantiated information with the final goal of reaching healthy ageing.

## 5. Conclusions

The members of the *Sir2* family, known as sirtuins, have emerged as principal factors in regulating cellular response to stress, compromising conditions that have been directly linked to tumorigenesis and tumor development. The field of cancer metabolism is growing rapidly, and findings indicating that sirtuins can relate and interfere in a series of cellular and metabolic mechanisms leading up a new road to the exploration of potential cancer therapeutic targets. Several sirtuins play a dual role in tumorigenesis. This duality relates to SIRT6, and may result from tissue and tumor type-specific function. Evidence shows that SIRT6 overexpression or knockout in cells can result in different biological responses as tumor suppressor or promoter, depending on cell and tumor type. Therefore, depending on its biological mechanisms of action in each tumor, the inhibition or activation of SIRT6 might have therapeutic value. A deeper understanding of the biology of sirtuins, particularly of SIRT6 at both a molecular and physiological level will be essential for elucidating both the potential therapeutic benefits and the detrimental side effects of their activation or inactivation. Although there are still many unanswered questions regarding the roles of sirtuins in cancer, evidence indicates that this research area should remain of great interest. Regarding SIRT6, it would be of a great interest to further characterize which cancer type requests SIRT6 activation or repression in order to effect tumor growth, as well as to define the regulated pathways unique to cancerous cells in order to translate this knowledge to specific targeting of this enzyme, leading to a promising new strategy in cancer therapy.

## Figures and Tables

**Table 1:** SIRT 1–7 characterization: size, localization, enzymatic activity and function.

Sirtuin	Size	Localization	Enzymatic Activity	Function
SIRT1	82 kDa	Nucleus	Deacetylase	Glucose production, insulin secretion, fatty-acid mobilization/oxidation (liver/skeletal muscle), cholesterol regulation, adipokine regulation, neuroprotection, stress resistance, apoptosis control, cell differentiation, mediation of calorie restriction
SIRT2	42 kDa	Cytosol	Deacetylase	Tubulin deacetylation, cell cycle control
SIRT3	44 kDa	Mitochondria	Deacetylase	Thermogenesis/metabolism, ATP production, mitochondrial fatty-acid oxidation
SIRT4	35 kDa	Mitochondria	ADP ribosyltransferase	Insulin secretion
SIRT5	34 kDa	Mitochondria	Deacetylase	Urea cycle regulation
SIRT6	39 kDa	Nucleus	ADP ribosyltransferase	DNA repair, telomeric chromatin structure, *NF-κB* regulation, metabolism
SIRT7	48 kDa	Nucleolus	Deacetylase	rDNA transcription
